# “It Would’ve Been So Beautiful…If the Hospital Didn’t Have to Tell the Police”: The Incompatibility of Mandatory Reporting Policies and Adolescent Survivors’ Post-Assault Needs

**DOI:** 10.3390/bs16010149

**Published:** 2026-01-21

**Authors:** Jessica Shaw, Caroline Bailey, Abril N. Harris, Megan R. Greeson, Anastasiya Danylkiv

**Affiliations:** 1 Department of Psychology, University of Illinois Chicago, Chicago, IL 60607, USA; 2 School of Social Work, University of Washington, Seattle, WA 98105, USA; 3 Department of Psychology, DePaul University, Chicago, IL 60614, USA

**Keywords:** sexual assault, mandatory reporting, adolescents, help-seeking, reporting

## Abstract

Adolescent sexual assault survivors have myriad post-assault needs. However, if and how they access resources to attend to those needs can be complicated due to their legal status as minors and child abuse mandatory reporting policies. Such policies that require specific entities to be notified when a sexual assault involving a minor has occurred might deter adolescents from seeking post-assault care. However, no studies to date have examined how mandatory reporting laws inform adolescents’ post-assault decisions and experiences. Through semi-structured qualitative interviews with twenty-one survivors of adolescent sexual assault in one Northeastern US state, we found that mandatory reporting actively deterred sixteen survivors from seeking formal help; would have deterred two survivors from seeking formal help had they known about it; and was a nonissue for three survivors who chose to seek formal help in an attempt to have very specific needs met. Survivors of adolescent sexual assault had serious concerns about losing agency and control and about unwanted involvement from police, parents, and child protective services. Individual providers, organizations, and whole communities must seriously consider the potential harm of mandatory reporting policies and think creatively and collaboratively alongside adolescent survivors to ensure they can access the care they need and deserve.

## 1. Introduction

Adolescent sexual assault (ASA) is far too common. In the United States, one third of adults who have experienced unwanted sexual contact were first victimized when they were between 11 and 17 years old. Indeed, recent national studies have found that over one-in-ten high school students reported sexual victimization in the past year alone, with higher rates of victimization reported among female students (18%); lesbian, gay, bisexual, and questioning students (17–25%); and transgender students (32%; Clayton et al., 2023; Lowery St. John et al., 2023). Like all survivors of sexual violence, adolescent survivors have varied post-assault needs. However, if and how they access resources to attend to those needs can be complicated due to their legal status as minors and child abuse mandatory reporting (MR) policies. Specifically, some MR policies require service providers to report any incidents of sexual violence in which a minor was victimized to specific entities. While an adult survivor can choose to engage specific providers for specific services in a confidential manner (e.g., to access post-assault medical care for a medical forensic exam without engaging law enforcement), an adolescent survivor’s choice to engage a single provider may result in the notification and engagement of additional providers and entities, whether or not this response aligns with the survivor’s wishes. Prior research documents the importance of control, privacy, and confidentiality for adolescent survivors (see Bailey et al., 2023, 2024 for reviews). However, there is a dearth of research focusing specifically on the role and significance of MR policies for teen survivors. As part of a larger mixed-methods project examining the impact of MR on ASA survivors and criminal cases, we interviewed 22 survivors who were sexually assaulted between the ages of 12 and 17 years old to examine the significance of MR policies in their post-assault decisions and experiences. This manuscript reports on what we found and makes a substantial contribution to our understanding of MR responses in the context of ASA.

### 1.1. The Ecology of Survivor Help-Seeking

Adolescent survivors’ post-assault needs are multi-faceted; survivors may need medical care, services that help ensure their immediate and long-term safety, a means to hold the person who harmed them accountable, and assistance in understanding what resources are available and how to best navigate through complex systems to obtain them. Adolescent survivors’ decisions on how to have their unique needs met occur in a broader ecological context. Kennedy et al.’s (2012) conceptual model of the help attainment process demonstrates that decisions whether and how to seek help are a complex interplay of individual, institutional, community, and societal factors. In deciding whether to seek help, survivors assess their own needs (e.g., do I need medical care?), whether they believe available resources are able to meet them (e.g., will the hospital actually be able to help me?), and how the perceived costs of seeking help relate to the anticipated benefits (e.g., will my parents finding out just disrupt my life even more?). The survivor’s social location and developmental context, their perceptions of and prior experiences with the institutions they are considering obtaining help from, and other community and societal factors all influence this assessment and their decisions whether and how to seek help.

### 1.2. Adolescents’ Post-Assault Engagement with Formal Systems

A limited body of research finds that most adolescent survivors choose not to report their assault to the police or seek post-assault medical care. Surveys with nationally and statewide representative samples reveal that only 8–30% of adolescent assaults were reported to police (Casey & Nurius, 2006; Finkelhor & Ormrod, 1999; Finkelhor et al., 2011; Kilpatrick et al., 2003). Though recent research on receipt of post-assault medical care among adolescents is particularly scarce, studies suggest that most teen survivors do not choose to seek post-assault medical care. When they do receive such care, often more time has passed since the assault as compared to older survivors; they receive fewer acute care services than older survivors; and receipt of care is sometimes a result of adults in their lives deciding such care was necessary (Campbell et al., 2015; Du Mont et al., 2016; Finkelhor et al., 2011; Jones et al., 2003; Muram et al., 1995; Peipert & Domagalski, 1994).

Consistent with Kennedy et al.’s (2012) conceptual model of help attainment, early research on general help-seeking among teens finds that adolescents may be reluctant to engage with formal response systems (e.g., police or healthcare providers) because they are concerned about confidentiality and privacy (Dubow et al., 1990; Helms, 2003; Kuhl et al., 1997; Schonert-Reichl & Muller, 1996). Though all survivors may have confidentiality concerns, they can be particularly salient for adolescents (Finkelhor & Wolak, 2003; Finkelhor et al., 2001). Adolescent development is marked by an increased identification with peers, as well as an assertion of autonomy and independence from adult interference (see also Bailey et al., 2024; Finkelhor & Wolak, 2003; Finkelhor et al., 2001). Teens who engage formal response systems may be teased, stigmatized, or ostracized by their peers; this may be perceived as a greater price to pay than the actual victimization, introducing an important factor for teens when deciding whether to report or seek formal help (Finkelhor et al., 2001; Jaffe et al., 2022; Miller et al., 2011). An adolescent’s desire to be independent from adults and authority figures—who often act as gatekeepers to post-assault resources and system services—may further inform the teen survivor’s decisions whether to report to police or visit the hospital. In one of the only studies to interview adolescents about their experiences seeking and attaining post-assault care, Campbell et al. (2015) found that adolescents attempted to exercise agency and maintain control over how much different individuals and entities knew about their assault. This suggests that even if confidentiality concerns do not deter initial contact with formal system personnel, they may remain a major concern throughout the help-seeking process.

Research on general help-seeking among teens shows that adolescents have clear ideas about what will be helpful or unhelpful to them (Helms, 2003). While adolescent survivors may seek guidance from the adults around them, they also prefer control over their post-assault help-seeking decisions (Campbell et al., 2015). However, adolescents may or may not be given a choice. Adolescents who do want formal help can sometimes seek it independently, without their parents’ consent (e.g., the right to consent to a medical forensic exam is often extended to individuals under the age of 18 years old). However, adolescents’ limited rights mean that those who do not want to seek formal help can have these preferences overridden by authority figures (e.g., parents/guardians, systems personnel) who learn about the assault. For example, parents and other guardians may force or coerce adolescent survivors into seeking help against their will (Campbell et al., 2015). Furthermore, adults or systems personnel may be legally mandated to report the assault to police regardless of the adolescent’s wishes.

### 1.3. Mandatory Reporting and Adolescents’ Post-Assault Decisions to Access Formal Systems

Child abuse MR policies, and corresponding practices, vary from state to state in regard to specific incidents that mandate a report and the agencies involved in the MR response (see Bailey et al., 2023 for a review). Even within the same state, there is variation in how individuals and organizations understand and carry out their MR responsibilities. The shared defining feature of such policies is that certain individuals are legally mandated to report suspected child abuse and neglect to select entities, regardless of whether the individuals harmed in said incidents want a report made. The entities to which these reports must be made are most often those tasked with protecting the harmed individual (e.g., child protection services) or holding the person who harmed them responsible (e.g., police and prosecution).

The extent to which MR policies apply to incidents of ASA varies significantly across states based on their laws and even within states based on how providers interpret them. Of particular importance is how ‘child abuse’ is legally defined and understood and if ASA (always) meets this definition. In some states, the legally defining feature of ‘child abuse’ is the age of the person harmed; thus, all incidents of sexual violence targeting a minor, regardless of other circumstances of the assault, would require a report under MR laws (e.g., see Arkansas Child Maltreatment Act §§ 12-18-103). In other states, additional criteria must be met for child protection services (CPS) to accept a mandated report; such criteria often relate to the relationship between the person harmed and the person who harmed them, such that the latter must be a family member, caregiver, or person in a position of trust or authority (e.g., see Illinois Abused and Neglected Child Reporting Act 325 ILCS 5).

While MR policies have existed since the 1960s, little is known about their impact (Bailey et al., 2023). And while these policies are often applied to incidents of ASA, only one study exists that has reported findings that relate at all to MR policies in this context. Using data from the 1995 National Survey of Adolescents, Stein and Nofziger (2008) examined to whom adolescent survivors disclosed and how this influenced the likelihood of an arrest. Of the cases in which adolescents chose to disclose to someone, only 7% were reported by the adolescent to a mandatory reporter (i.e., police, social workers, doctors, teachers, principals, and school nurses). “The actual number of adolescents who told mandatory reporters was very small,” and led to only a few arrests (Stein & Nofziger, 2008, p. 167).

Beyond this single study, we do not know how MR intersects with adolescents’ post-assault decision-making. Given adolescents’ concerns about privacy and confidentiality when choosing whether to seek help, we might anticipate that MR acts as a deterrent to care and support for teen survivors, as it requires the notification and engagement of a range of entities, regardless of the individual survivor’s wishes. Even if MR does not deter adolescent survivors from seeking post-assault care, perhaps because they are unaware of MR policies, it may be upsetting for teens to learn at the time of receipt of care and system contact that their expectations regarding confidentiality and next steps may not be realized. However, it is also possible that other essential factors inform adolescents’ post-assault decisions whether to access formal support and service systems, such that MR policies do not play a significant role. For example, adolescents who choose to seek post-assault medical care may be most concerned with getting their healthcare needs met and are not concerned about CPS or other entities being notified. Regardless, to help ensure adolescent survivors are able to access services and supports that best attend to their myriad post-assault needs, we need to understand how MR impacts teens’ post-assault decisions.

### 1.4. Current Study

Kennedy et al.’s (2012) conceptual model emphasizes the complex interplay of individual, institutional, community, and societal factors in survivors’ decisions whether to seek help. Survivors assess their needs, available resources, and potential costs as they decide to seek, or decide not to seek, specific types of help. However, prior to this study, minimal research has explored adolescents’ post-assault decision-making and experiences with responder systems (see Campbell et al., 2011); no prior research had focused specifically on understanding these experiences in relation to MR. This presents a significant knowledge gap in which we do not know if MR is helping ASA survivors by connecting them to services that can interrupt and respond to harm, or if MR is preventing adolescent survivors from accessing much-needed care. Indeed, the larger mixed-methods project in which this study was embedded was designed and implemented in response to the informational needs of a statewide multidisciplinary adolescent taskforce (Shaw et al., 2022; see also Shaw et al., 2024). Initially convened by a Sexual Assault Nurse Examiner (SANE) program in one Northeastern state, this taskforce consisted of representatives from many different agencies and professions that respond to ASA survivors. The taskforce set out to review the current response system to develop best practice guidelines for responders. There was a particular focus on the response to adolescents who initially encountered formal help systems when they sought immediate post-assault medical care. SANEs are specially trained to provide comprehensive, expert medical forensic care and were often adolescent survivors’ first point of contact with formal help systems. The SANE program wanted to ensure they understood the roles, practices, and coordination among the many different entities involved in responding to ASA so they could explain to their patients what they could expect to happen after receiving care. The SANE program’s policy, informed by state legislation, required the SANE to complete a mandatory report to CPS for all patients under the age of 18 who they treated post-assault. Upon receipt of the report, CPS would screen the case to determine if it warranted a CPS response. As part of the screening and response determination process, CPS would engage the adolescent’s parents and oftentimes the person who harmed them. Regardless of the screening outcome, state mandatory reporting laws also required CPS to forward the case to the police and prosecutor’s office. Taskforce members discussed and wanted to know more about how this process impacted adolescent survivors’ post-assault decisions and experiences.

Accordingly, to attend to this knowledge gap in the literature and the informational needs of our community partners, we employed a pragmatic approach to learn from survivors of ASA what role and impact MR played in their post-assault decision making on whether to seek formal help. A pragmatic approach directs those who use it to seek practical and useful answers that can provide direction in addressing concrete problems (Creswell & Poth, 2018; Patton, 2014). Pragmatism requires researchers to choose methods, techniques, and procedures based on what will best answer their research question(s) while engaging in reflexive practice that attends to the social, historical, and political contexts in which the researcher exists and the research occurs. Consequently, we set out to ask and answer the following question: what is the role and impact of MR in adolescent sexual assault survivors’ post-assault decisions on whether to seek formal help? Our research team’s decision to ask this question, and to center survivors’ voices in answering it, demonstrates our team’s commitment to serving survivors and using research to improve how systems respond to them. All decisions throughout the research process, then, were guided by this ethos and the absolute necessity of scientific rigor to ensure we examined how MR impacted survivors—for the better or worse, or not at all. At the beginning of this project, and routinely throughout it, our team made explicit and discussed our existing and emergent values, beliefs, and experiences related to the research focus so that we could interrogate them directly, disentangle them from the research, and make intentional decisions about our collective work. Many of these strategies, including extensive memoing, consensus meetings, and systematically searching and accounting for confirming and disconfirming evidence for each finding, are woven into and appear in the Materials and Methods section, below.

## 2. Materials and Methods

### 2.1. Sample and Recruitment

Our sample included survivors of ASA who were sexually assaulted in the focal state when they were between 12 and 17 years old. This age range included survivors that could access SANE services without a parent or guardian (i.e., 12 years old and older) and to whom the MR response would apply (i.e., 18 years old and younger). Participants could be any age at the time of the interview and could have experienced multiple victimizations in addition to the sexual assault that made them eligible to participate (hereafter, “focal assault”).

Modeled after Campbell et al.’s (2011) approach to recruiting adolescent survivors, we employed two recruitment strategies. First, we partnered with one hospital and the SANE program to introduce the study to eligible patients at the time they received post-assault medical care from the SANE. If patients indicated they were interested in learning about the study, a research team member contacted them 2–3 weeks after their hospital visit to provide them study information and schedule an interview. This recruitment strategy allowed us to recruit survivors who we knew encountered the MR response immediately post-assault. From July 2018–November 2019, SANE treated nine eligible patients at the partnering hospital. The study was introduced to six of these patients, three of whom agreed to be contacted, and two of whom ultimately participated. We were unable to reach the third patient who agreed to be contacted, speaking only with their parent, who stated they were too young to participate in any research.

Our second recruitment strategy involved partnering with local rape crisis centers, domestic violence agencies, and other community organizations that provided SA-specific services to make the opportunity to participate available to a wider range of survivors, including survivors who chose not to seek post-assault medical care. We provided partnering agencies with paper flyers and specific information to post on social media platforms. While survivors could have been sexually assaulted anywhere in the focal state to be eligible to participate in this study, we partnered with service providers in the same city in which the hospital was located, which allowed us to routinely visit partners’ sites to restock recruitment materials. Partnering agencies made information on the study available from August 2018–November 2019. During this time, 34 potential participants made initial contact with the research team. Twenty of these individuals were ultimately interviewed. Fourteen individuals were not interviewed either because they did not meet our inclusion criteria (*n* = 3), because we were not able to reach them after initial contact (*n* = 2), or due to scheduling or transportation challenges (*n* = 9).

We interviewed *N* = 22 survivors. This exceeded our initial target sample size of *N* = 20, initially set with a goal of achieving data saturation (Bernard et al., 2017; Hennink & Kaiser, 2022). While recruitment ceased once we reached our target sample size (i.e., we removed all recruitment materials and instructed SANE to stop introducing the study), we carried out all interviews with eligible participants who had already seen our materials and contacted our team. At the time of the interview, participants ranged in age from 15 to 55 years old. Between a few weeks and 41 years had passed since the focal assault. At the time of the focal assault, participants ranged in age from 12 to 17 years old, with half of the participants disclosing additional SAs that occurred before, during, or after the target age range. Only one participant was sexually assaulted by a stranger during the focal assault; all other participants (*n* = 21) were assaulted by someone they knew, including friends, acquaintances, intimate partners, and family members. Most focal assaults involved a single perpetrator (*n* = 18), with the remaining focal assaults involving multiple perpetrators (*n* = 3), or the participant being unsure of how many perpetrators were involved (*n* = 1). Following the focal assault, three participants received immediate post-assault medical care from a SANE and encountered the MR response, two participants interacted with CPS, and nine participants had their focal assault reported to police. Participants were asked how they would describe their race or ethnicity and their gender. Most participants self-identified as white (*n* = 15), with one of these participants identifying as white Ashkenazi. The remaining seven participants identified as Black (*n* = 4; “African American,” “Black,” “American descendant of slaves”), Black and Puerto Rican (*n* = 1), Hispanic or Latino (*n* = 1), or Korean, Asian mixed (*n* = 1). The majority of participants also self-identified as women (*n* = 16; “girl,” “female,” “cisgender female,” “woman,” “cis female,” “she/hers”). One participant identified as male, and five participants identified as transgender or gender-expansive (“non-binary,” “non-binary, more female,” “presenting as a woman but still figuring it out,” “gender-fluid non-binary,” “trans-masculine”).

### 2.2. Data Collection

Interviews were conducted by three members of the research team who identified as women or were femme-presenting (the PI and two research assistants). All interviews were conducted in person at a rape crisis center with a rape crisis staff member available should the participant become distressed and need to connect with them. We never needed to make use of this resource during the interview, but we often connected participants with staff members after the interviews if they wanted to learn more about available services. Prior to the start of the interview, participants were guided through the informed consent process and provided with a USD 40 stipend. To protect the privacy and identity of all participants, we obtained IRB approval for a waiver of documentation of informed consent, as the consent form would be the only remaining record of an individual participant’s identity after their interview was de-identified and analyzed. In line with our IRB-approved protocol, we sought informed assent from participants under the age of 18 years old, and consent from their parent or guardian. Participants were encouraged to prioritize their comfort and were informed that they could take a break or stop the interview at any time. The research team also provided drinks, snacks, and fidgets. Data collection proceeded with a semi-structured interview guide that probed six areas: (1) their decision to participate in the study; (2) background on the assault; (3) disclosure and post-assault medical care decisions; (4) post-assault experiences with system personnel; (5) recommendations for system personnel and the interviewer; and (6) demographics. Interviews lasted between 49 and 151 min (*M* = 95 min, *SD* = 30 min) and were recorded with the participant’s permission. Post-interview, we provided participants with information on available resources and offered to connect them with the rape crisis center staff member to learn more.

### 2.3. Data Analysis

Interviews were transcribed, reviewed for accuracy, de-identified, and entered into NVivo 13 software for qualitative data analysis. We used Miles et al.’s (2020) three-phase data analysis process, consisting of data condensation, data display, and conclusion drawing and verification. Though listed here as three separate phases, they overlap and are less distinct in practice. The first phase, data condensation, includes reading, memoing, organizing, and coding the transcripts. Five members of the research team (the PI and four research assistants, three of whom also conducted interviews) independently read and generated analytic memos for each transcript (see Bernard et al., 2017; Creswell & Poth, 2018). The research team then discussed their memos to develop a shared understanding of the scope and content of the dataset and to develop an analytic outline clearly delineating the foci for the next step in analysis (i.e., coding, see Tracy, 2013). Two members of the research team (who also conducted interviews) then independently coded one-third of the sample inductively (*n* = 7 transcripts), focusing on coding the concepts most related to the focal research question of the significance of the MR response in relation to ASA survivors’ post-assault decisions. This included coding inductively for concepts related to survivors’ decisions to disclose or seek care. The codes applied to the first seven transcripts were then reviewed, sorted, and organized to create an initial codebook which was applied to the remaining transcripts. Each remaining transcript was coded by two independent coders. The codes were compared, disagreements were flagged, and the research team met to review and discuss disagreements until consensus was reached, updating the codebook as needed. The final codebook was then used to return to and recode the first seven transcripts employing these same steps (i.e., double-coded; discrepancy review; consensus meetings).

Data display, the second phase in Miles et al.’s (2020) three-phase process, consists of creating organized visual displays of the data that will aid in making sense of them. While coding, the research team created a series of data displays presenting participant demographics and histories; focal assault characteristics and summaries; the extent of system/responder engagement; MR summaries; disclosure process summaries; and specific MR concerns. Data displays were also used to organize codes and themes. We used these data displays to draw and verify conclusions by noting patterns and themes, making contrasts and comparisons, and accounting for confirming and disconfirming evidence for each finding (see Miles et al., 2020). One participant who chose not to have their interview recorded was excluded from this detailed analysis, yielding a final analytic sample of *n* = 21.

## 3. Results

**Figure 1:**
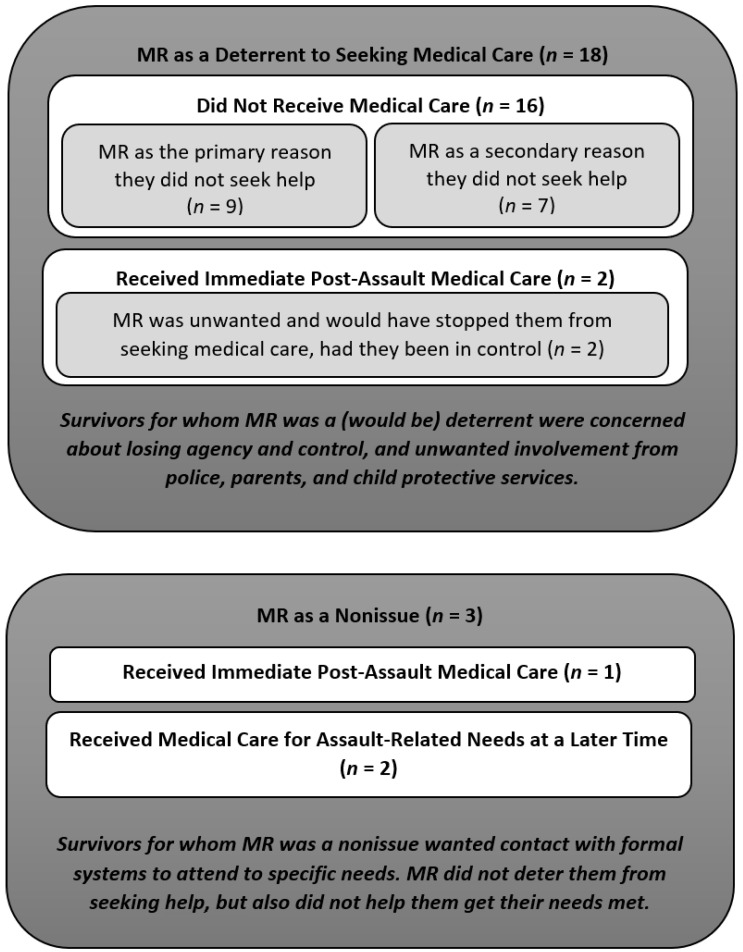
How mandatory reporting influenced survivors’ decision making.

We were interested in understanding how the MR response impacts adolescent survivors’ post-assault decisions on whether to seek help. Of the twenty-one interview participants, the MR response actively deterred, or would have deterred, eighteen participants from seeking formal help. For the three remaining interview participants, the MR response did not deter them from disclosing and engaging formal systems for help. However, it was also of no assistance to these survivors in obtaining the specific help they sought. Table 1 presents descriptions of each participant and the way in which the MR affected them, whereas Figure 1 summarizes all findings.

### 3.1. Mandatory Reporting as a (Would Be) Deterrent (n = 18)

MR acted as a deterrent, or would have been a deterrent, for eighteen of the twenty-one interview participants. These participants were worried about others whom they did not want involved or notified about their assaults, particularly the police, their parents, and, to a lesser extent, CPS. Participants in this group were also concerned about losing control over what would happen next if, or when, they initiated the help-seeking process. As detailed below, the exact role of MR in their decision-making process varied across the participants.

#### 3.1.1. MR as a Primary or Secondary Deterrent to Seeking Help (*n* = 16)

Sixteen participants did not seek formal help, with MR as a key factor in their decision. For 9 of these 16 participants, MR was a primary deterrent. These survivors considered seeking formal help but chose not to because they were concerned that formal help-seeking would lead to others finding out about their assault and them losing control over the situation. For these survivors, knowing or fearing that others would be contacted was the main (or only) reason they did not seek post-assault care. For the other seven participants in this group, MR served as a secondary deterrent (see Figure 1). These survivors had other primary reasons they did not want to seek post-assault care. They explained how they did not think their assault was serious enough, they did not think post-assault services would help, or they did not recognize their experience as sexual assault until later. While these were the primary barriers to seeking formal care, they, too, discussed concerns about others finding out and the resulting loss of control. For these survivors, MR was not the primary deciding factor, but it still served as an additional, secondary deterrent to seeking help. Despite these differences in whether MR was a primary or secondary deterrent, it contributed to their decision to not seek care, with this group of survivors sharing concerns related to unwanted police involvement, parents finding out, and losing agency and control over what happened to them next.

***Unwanted police involvement:*** For participants for whom MR was a primary or secondary deterrent, concerns that police would be notified as part of seeking formal help were a key consideration. Many of these survivors found the prospect of involving police to be particularly upsetting. They discussed how they “were really worried…that I’d have to tell the police,” and “wasn’t ready for that,” (Participant 139, MR as primary deterrent), how “just imagining it is too uncomfortable” (Participant 117, MR as secondary deterrent), and if they had gone to the hospital and learned that police would be notified, “that would have been miserable” (Participant 141, MR as primary deterrent).

Survivors described how they were dissuaded from seeking help that would result in police notification because they believed the police would not help them, and would instead cause harm. For example, Participant 126 (20 y.o. Korean, Asian, mixed transmasculine person) was sexually assaulted by a romantic partner when they were both 13 or 14 years old. MR was a primary deterrent for this survivor. The participant did not go to the hospital because of fears that the hospital would call the police and the police would not help.

“If I have to tell the hospital, then maybe I have to tell the police. And I’ve had other friends who have gone to the police for assault and nothing came of it. And, so, you know, all it would do would tell my assailant that I told somebody and then I would be in danger. And nothing would come out of it, other than that.”

Participant 126 went on to explain how he was “very scared of police interaction” because his assailant would find out he “told somebody and they told me not to tell anybody.” Participant 126 also implied a fear of negative legal consequences for himself, as he was “doing some illegal things” that he “didn’t really want the police to know about.”

MR was a secondary deterrent for Participant 123 (19 y.o. Caucasian who presented as a woman but was “still figuring stuff out”) was sexually assaulted when they were 17 years old by a 19-year-old friend of a friend. They chose not to seek post-assault medical care primarily because they didn’t think they would be taken seriously. But they also discussed how the potential for police involvement deterred them, and likely other survivors, from seeking help.

“I definitely think that a police presence [post-assault] would make many people, myself included, extremely uncomfortable, and 99 times out of 100, they’re just not gonna do anything besides make it worse. And if you’re going to be vulnerable and seek help for something like that, you want it to be someone who is informed and knowledgeable and that’s pretty much their only job…The [policing] profession doesn’t really attract people who are into conflict resolution. I’m not super comfortable around guns and I’m not personally at high risk of police brutality, but it definitely happens all the time and that’s just added anxiety into a situation where you are vulnerable in seeking help…I really wouldn’t go to the police unless it was a situation where I’m in continued danger from this person. And even then, there are just so many cases of women going and reporting a stalker or an abuser and just being laughed off.”

While police may not respond in person at a hospital unless the patient requests it, the possibility of their involvement contributed to Participant 123’s decision not to seek post-assault medical care. Other survivors felt similarly. Participant 129 (35 y.o. Black cisgender woman) was sexually assaulted by her 17-year-old romantic partner when she was 15 years old. She, too, was primarily dissuaded from seeking help due to MR; it was a primary deterrent. She explained how she has “always been sort of weary of police…[as they] don’t have a great reputation with people of color.” She “just grew up not trusting the police were ever here to serve me. They were here to serve white people and rich people and not me.” For Participant 129, the historical and contemporary racism exhibited by police seemed to influence her decision not to involve them.

***Fear of parents finding out:*** Interview participants also described how they were “scared,” (Participant 129, MR as primary deterrent) “afraid,” (Participants 108, MR as secondary deterrent; 111, MR as primary deterrent; 115, MR as primary deterrent) or “terrified,” (Participants 108, MR as secondary deterrent; 129, MR as primary deterrent) of their parents being notified. This was often the case for participants for whom MR was a primary deterrent. For example, Participant 136 (37 y.o. woman and American descendant of slaves) was sexually assaulted by a 20-something-year-old acquaintance when she was 14 or 15 years old. Participant 136 explained that she never considered going to the hospital for care because she knew care providers “were very big on parental consent. Anything that happened to a minor…your momma gotta get called.” Participant 136 explained that her mom being notified about the assault “would be the most devastating thing…[and I would make sure] that was not happening in my situation.” When asked what would have happened if she had gone to the hospital for care and was told her mom would be notified, Participant 136 explained that “I would have left. Straight up. I would have been, ‘can I use the bathroom?’ and I would have bounced.”

Even participants for whom MR was a secondary deterrent discussed how parental notification or engagement was incompatible with what they would have wanted to happen if they had sought care. For example, Participant 108 (25 y.o. white cisgender female) was sexually assaulted by different classmates at different parties when she was 16 years old, and again when she was 17 years old. She explained how she chose not to go to the hospital primarily because she did not initially acknowledge her experience as an assault. As she reflected on her decision during the interview, she explained that if she had recognized her experience as an assault, she still would not have gone to the hospital for fear of her parents finding out.

“I didn’t think what I’d experienced was assault, so I didn’t think that going to the hospital would even make sense. I think if I had known that [it was sexual assault], I don’t think I would’ve gone to the hospital because I would’ve been so terrified of my parents finding out, (a) whatever happened, (b) that I was drinking, (c) that I was doing these things. I would’ve been so terrified of the reactions from my parents…that I wouldn’t have gone even if I had been—what in my mind rape was—would’ve been violently raped…as like what you see in [the television show] SVU…[that’s] what I would’ve thought that rape was…Even if I was SVU raped, air quotes, I still wouldn’t have wanted to go to the hospital because I would’ve been so afraid of what my parents said.”

Participant 108 made clear that even if she needed medical care, she would not have sought it out because it would mean her parents would have found out about her assaults. At the time of her interview, Participant 108 remained firm in her decision not to disclose any of her teenaged assaults to her parents.

Other participants for whom MR was a primary or secondary deterrent explained that they, too, “would not have talked to them [their parents] about this [assault] at this age [of adolescence]” (Participant 111, MR as primary deterrent); that their parents finding out “would put me in a situation I didn’t wanna be in” (Participant 141, MR as primary deterrent); and how their parents finding out about their assaults would “paralyze” them (Participant 128, MR as secondary deterrent). Many participants in our sample stated plainly that the possibility that “they’d have to tell someone” was the “driving force of [why] I didn’t want to go and get service[s]” (Participant 139).

***Loss of agency and control:*** In addition to sharing specific concerns about MR leading to unwanted involvement with additional entities like police and parents, participants also described a more generalized fear of not being allowed to make decisions and of losing control over what would happen next. This was discussed for both survivors for whom MR was a primary and secondary deterrent, but was particularly salient for the primary deterrent group. For example, Participant 130 (29 y.o. white female) was sexually assaulted by her 16-year-old romantic partner when she was 13 years old. For her, MR was a primary deterrent. She explained how she was aware of MR policies, and how they miss the mark when it comes to meeting survivors’ needs.

“If somehow, I could’ve just got help and my whole life wouldn’t crumble from it, and my parents weren’t told about it, it would’ve been so beautiful. And, I know that there are laws…when you’re underage, parents have to get involved and everything. But sometimes that’s not the best. And it [should be] the victim’s choice on when people find out about stuff. It doesn’t matter who it is in their life, if they wanna keep the secret from somebody, they have the right to do that…I know that, as a minor, I had no rights. So you’re stuck in this loop of, ‘well, I’m just gonna sit up and shut up, and take all of the abuse.’ So then it just fed into the cycle of keeping quiet. And just figuring out ways to keep quiet and hunker down. [Otherwise], once you try to take care of yourself, this whole process kicks in and it’s overwhelming and scary and too fast.”

Participant 130 went on to explain why it is so important for survivors to have the power to make decisions rather than MR dictating what happens next.

“Something was taken from them already. So they have an example where you are powerless. People and things can take pieces of you, from you, and they need to get out of that mindset as quick as possible. Because if you’re not taken out of that mindset as quick as possible, it would be a cycle that continues their whole life. And they will be a disenfranchised soul forever. And by having that teenager have the power over how they reach out for help and what resources are available, it’s stopping the repetition of losing your voice and losing a piece of you.”

Participant 130 was not alone in this sentiment. Even when participants explained how they were not aware of, or did not understand, MR policies, they chose not to seek help because the risk of losing control was too great. Participant 139 (23 y.o. white cis-female) was repeatedly sexually assaulted by a live-in family friend starting at the age of 13 years old and was sexually assaulted by a family member when she was 15 years old. Participant 139 describes how she considered telling someone about her assaults around the time they were happening, what ultimately informed her decision not to do so, and how she feels about that decision today.

“There was a couple of people when I was younger that I got really close to telling. The first one was my school social worker. I really, really liked her…I don’t know why I never did. I think I was maybe afraid that she’d tell my mom, or that she would have to call the police, you know I didn’t understand mandated reporting or anything like that. I think I’m still happy with that decision not to tell her because if she had reported, I don’t think I was ready to handle what [would have] happened.”

Participant 139 went on to explain why being denied agency and losing control of the situation is so harmful, particularly for someone who is sexually assaulted when they are a teenager.

“I wanted to be able to tell someone and then have the choice of what I wanted to do with that information. And to be honest, if I was given the choice, I probably would have chosen the thing they wanted. I probably would have gone to the police, or the hospital, or whatever, but I would have been more resistant if someone made me…I think when I was 13 to 15, those years, it had a lot to do with the fact that I was just a teenager that didn’t like to be told what to do…I was still discovering myself and you want everything to be your choice at that age, and your parents and teachers to stay out of your life…I think the other part of it is that so much of your decisions aren’t yours at that age…When you’re that young, once you tell someone that something happened to you, it’s not your body anymore. It’s your parents’. I don’t really know that many people who would have thought about it as my being and my body, because I was so young…I don’t think I was ready for people to think of my body and my experience as my mom’s, or the government’s, or anyone else’s…I wanted the power to make decisions. And I feel like if I’d told someone, I would’ve lost that power.”

Participant 139 explained how “someone else making the decision, without your consent or your agency involved, is gonna probably be more traumatic.” While MR was a primary deterrent for Participant 139 following her assaults as a teen, at the time of our interview she, herself, was now a mandatory reporter. Participant 139 explained that “it’s really shitty.” When Participant 139’s clients “tell me something that personal, I so know that they need more time, but I don’t have a choice. And it’s really hard to do that, and then watch them feel really, really betrayed.”

Like Participant 139, other participants in our sample intentionally delayed disclosing what happened to them until they were older. This was because they could retain control over what happened next, as the person in receipt of their disclosure “couldn’t really do anything about it because I was already 18” (Participant 141, MR as a primary deterrent).

#### 3.1.2. MR Would Have Deterred Them: The Unexpected, Unwanted MR Response (*n* = 2)

For the sixteen participants in the previous group (MR as a primary or secondary deterrent), MR caused or contributed to their decision to not seek care. For two other survivors, MR would have been a deterrent, but they still received post-assault care (see Figure 1). These two survivors were not aware of MR when they made decisions to initiate the help-seeking process. This led to their receipt of post-assault medical care, and MR, which was not in line with their wishes. Both survivors were clear that if they had been fully aware of MR, they would have made different decisions that would not have put into motion, or otherwise terminated, the MR process. In other words, MR would have been a deterrent to seeking post-assault medical care, but they were not fully aware of how it worked and at what point they would be disallowed from making decisions. These survivors feared, and sometimes experienced, unwanted parent and CPS involvement and loss of agency and control.

***Unwanted parent and CPS involvement:*** Participant 107 (16 y.o. African American female) was not able to avoid unwanted involvement of others via MR because a friend reported the assault to a mandatory reporter. Participant 107 was repeatedly sexually assaulted by a relative’s live-in boyfriend from the ages of 9 to 13 years old. Participant 107 initially disclosed what was going on to a friend at school, who then told a teacher, which initiated the MR response. The teacher told the guidance counselor, who notified Participant 107’s mom that something had happened, but without additional information, leaving it to Participant 107 to detail what had happened. Participant 107 told her mom, who told other members of the family, and took Participant 107 to the hospital for a medical forensic exam, then to the police station to make a report. This sequence of events—from the initial disclosure to her friend, to having to talk with the teacher, her guidance counselor, parents and family, to going to the hospital and police station with her parents—unfolded in a single day. Participant 107 described initial concerns about her parents finding out, “you just can’t tell your parents that you went through something like that.” Participant 107 explained that once the process started, “the main thing I was concerned about” was CPS being notified and “being taken away from my house.” This did not happen, and at the time of our interview, Participant 107 was satisfied with the current state of the ongoing criminal case that resulted from the harm she had experienced. Still, Participant 107 shared in her interview that when this all was transpiring, she “woulda pulled the plug on the whole thing” if she could.

Similarly, Participant 138 (16 y.o. Black and Puerto Rican female), who was sexually assaulted by multiple people at a friend’s party when she was 16 years old, did not know about MR and how it would play a role in her post-assault care experience. She chose to go to the hospital after her assault and learned at the time of receiving care that a report would be made to CPS. She explained that had she known that ahead of time, she “probably [would] not” have gone to the hospital. In other words, the MR likely would have deterred her from seeking medical help, but she was unable to make that choice because she was not aware that a consequence of medical help-seeking would be unwanted CPS involvement.

***Loss of agency and control:*** Participant 107 (16 y.o. African American female), for whom MR was a would-be deterrent as she described how she “woulda pulled the plug on the whole thing” if she could, also discussed how quickly everything went and how little agency she had in the disclosure and MR response process.

“I just wish that people woulda give me a moment to breathe instead of going too fast, you know. Everyone was like, ‘okay, you can’t leave.’ I was like, ‘But what about if I take the day to breathe, take the school day to breathe for a minute, and then we could talk about this after school. And my mom can come after school so we can talk about it together.’ But then, ‘no.’ It’s like, ‘oh, let’s do this now.’…I wish people would give me moments to process it. ‘Cause everyone’s just so mad and crying, and I’m like, ‘I don’t understand. I need room to breathe.’”

Participant 107 felt like she “didn’t get a say on anything,” once the MR response was initiated and as though her “voice went unheard,” because others were making decisions on her behalf.

### 3.2. Mandatory Reporting as a Nonissue: Neither a Deterrent nor Helpful

For three participants, MR was a nonissue when deciding whether to disclose or seek formal help (see Figure 1). Each of these survivors had specific needs they attempted to have met by engaging formal supports, and they were not deterred by MR in doing so. All of these survivors also (eventually) chose to involve the police. Participant 101 wanted to know if what they had experienced was rape; Participant 119 wanted to be provided options for what they could do next; and Participant 118 wanted to secure a safe living environment by having their repeat perpetrator removed from their university dormitory. While MR did not deter any of these participants from engaging formal systems for help, it also did not facilitate receipt of the specific help they sought.

Participant 101 (16 y.o. African American female) was sexually assaulted at 15 years old by someone she met online. She described how she reached out to responders because she wanted to confirm what she experienced was rape. She “knew it was rape, ‘cause I did not want to have sex with him, but…kept denying it.” She called 911 to get the non-emergency police number as she “would like help,” but explained, “it’s not an emergency.” While she expected and hoped that “they were just gonna give me the number and [it would] be over with,” an ambulance was dispatched to her location. She agreed to go to the hospital for care as she was concerned police might otherwise “think I’m lying” and because “it didn’t feel like I had a choice.” Once at the hospital, the presumed MR response resulted in CPS presenting on site and talking with the survivor. While Participant 101 decided not to file a police report at the hospital, she contacted the police afterwards to make a report. Participant 101 was not concerned about different individuals or agencies being notified about what happened as part of the MR response as she had prior involvement with CPS. She did not want to describe any of her interactions with CPS but wanted the interviewer to record that they were “awful.” We interviewed Participant 101 a few months after the focal assault, and, at that time, she had not heard back from the police despite having reached out to them a number of times for updates regarding her case. Thus, while MR was a nonissue for Participant 101 when initially contacting police, the initial robust response from providers, in part dictated by MR policies, seemed for naught.

Participant 119 (35 y.o. white female) was sexually assaulted by a group of schoolmates at a party when she was 17 years old. While Participant 119 did not receive immediate post-assault medical care, she was seen by medical providers some time later after experiencing an array of physical and mental health impacts and after disclosing what happened to her mom. Participant 119 described how after explaining “what had happened to the doctors,” the doctors “didn’t wanna call it [sexual assault],” and that this experience “set the tone for a whole pattern of interactions with people.”

“Slowly people at the school found out. Teachers found out, coaches knew…the guidance counselor knew. All these mandated reporters knew and never reported…So I was very reluctant to engage with people after all those experiences where people [were] either shaming me for it or shushing me….And I think that’s really unfortunate because I think it would’ve helped me cope better and I would’ve been able to get justice better down the road, had mandated reporters done their job.”

When invited to reflect on what she wished those to whom she disclosed did differently, Participant 119 explained that she wished she “hadn’t felt so alone,” and that she “really would’ve liked to be connected to resources or people who had navigated this and survived it.” Participant 119 reported their assault to police some time later, after she went to college. While the criminal case went forward and resulted in convictions, MR was a nonissue in that it did not act as a deterrent or facilitator in Participant 119 connecting to much-needed resources.

Somewhat like Participant 119, Participant 118 was counting on and wanted others to do something. Participant 118 (21 y.o. white female) was sexually assaulted several times over a few days when she was 17 years old by a collegemate who lived in the same dormitory. Participant 118 turned 18 years old just a couple of days later. Following another threatening incident, Participant 118 disclosed to a resident advisor that they did not feel safe sleeping in their dorm room. Participant 118 chose not to go to the hospital post-assault as they did not initially acknowledge their experience as a sexual assault. “By the time I had realized I had actually been raped…it was…two months later. I was like, ‘well, what are they gonna do?’” Participant 118 described how they disclosed to many people, including formal responders, in an attempt to secure a safe living environment, separate from their perpetrator.

“At that phase of my life, I was literally [telling] anyone who will listen to me, please hear me. I don’t know if there’s anyone that I can think of that didn’t know. I think I was like, ‘someone just tell me I’m not going crazy. Someone please believe me.’”

These disclosures and attempts to obtain help, including to a dean, Title IX, and the police, were unsuccessful. Participant 118 eventually transferred to a new college and pursued a Title IX complaint with the support of her parents and a hired attorney. Like Participant 119, MR was a nonissue for Participant 118 as it neither deterred nor facilitated the actions she took to try to secure her safety. Figure 1 comprehensively presents the patterns observed in the role and impact of MR for all of the participants in this sample.

## 4. Discussion

To date, no prior research has directly engaged survivors of ASA to understand how MR impacts their post-assault decisions whether to seek formal care. In interviewing 21 survivors of ASA, we learned that MR actively deterred most of the survivors in our sample from seeking post-assault care. Of the few survivors who did receive post-assault care, they either would not have done so had they been in control or previously known about MR, or were so focused on attaining specific help that it did not affect their decisions. In this latter case, though, MR also did not help facilitate their needs being met. By demonstrating the unique needs and experiences of adolescents and the impact of MR on the survivors, this study reaffirms the importance of examining survivors’ help-seeking ecologically, with specific attention to the interaction of developmental and structural (i.e., policy) factors that influence help-seeking (Kennedy et al., 2012).

MR deterred, or would have deterred, eighteen survivors in our sample from seeking post-assault care. These participants were most concerned with police and parents being notified and involved in the response process. The specific MR response being examined in this study includes both entities. Of course, adolescent survivors may not be familiar with the particulars of the MR response; however, in our interviews, participants often explained how they understood at the time that “when you’re underage, parents have to get involved,” and that if only “the hospital didn’t have to tell the police,” they would have gone in for care (Participant 130, MR as primary deterrent). CPS is also a part of the MR response being examined in this study, and most commonly involved in MR responses across the United States. Participants for whom MR was a deterrent were concerned with CPS being notified to a lesser, though not insignificant, extent. Most survivors in our sample were so focused on the potential of police or parents being involved that they did not mention CPS at all. Those who did described concerns of “being taken away from my house,” (Participant 107, MR as would-be deterrent), or declining to discuss CPS in detail and instead asking that the interviewer be sure to record that they are “awful” (Participant 101, MR as nonissue).

Beyond being worried that specific entities would be notified about their assaults, study participants were deeply concerned about losing control of the situation and being denied the opportunity to make decisions that would significantly impact them. Participants highlighted the importance of “choice” and how so many “decisions aren’t yours at that age” (Participant 139, MR as primary deterrent). In disclosing their assault to others and seeking formal help, participants explained how that would mean that “it’s not your body anymore. It’s your parents’.” This fear of losing agency and control was shared not only by participants who chose not to seek formal help but also by those who did encounter formal systems. The three participants who received immediate post-assault medical care and encountered the MR response described concerns about not being able to control what was going to happen next. This aligns with Campbell et al.’s (2015) prior study in which they interviewed adolescent survivors who received formal help from the medical and legal systems; survivors in that sample discussed the importance of being able to exercise agency and retain control over who knew, and how much they knew, about their assault.

While a minority of our sample, MR was a nonissue for three survivors. These survivors had specific aims in engaging formal systems. Because they wanted providers to do something about their assault, they were not concerned about different entities finding out, and MR did not deter them from seeking help. However, MR was also of no service to them as it did not facilitate their needs being met. Thus, across all 21 participants in our analytic sample, MR did not help. This finding is particularly striking, as it was consistent for participants who were interviewed weeks after their focal assault, as well as those participants who were interviewed decades later. One might have expected that older participants, with more temporal distance from their assault, might look back and question their initial decision making. MR policies were created and implemented by adults who were in part concerned about the well-being of young people. Thus, older participants, with more lived experience as adults, more temporal distance from their assault, and looking back at their younger selves, might now see the value in MR, thus impacting how they recollect and recount their experience. However, this was not the case. Whether it had been a few weeks or 41 years since the focal assault, survivors in our sample consistently reported how MR prevented them from obtaining the care they may have otherwise pursued (i.e., primary deterrent), affirmed their decisions not to disclose or seek formal help (i.e., secondary deterrent), caused them distress upon learning about MR (i.e., would-be deterrent), or otherwise just did not help (i.e., nonissue).

### 4.1. Limitations and Future Research

While this project makes substantial contributions to our understanding of the significance of MR policies in ASA survivors’ post-assault decisions and experiences, it has important limitations. Our findings are based on interviews with twenty-one survivors of ASA in one study from one Northeastern state, thus limiting their generalizability. Generalizability is often discussed only in the statistical/probabilistic sense, where we assess the extent to which our findings come from a representative sample in such a way that they can be reliably generalized to a broader population (Smith, 2018). In this sense, our findings are not generalizable, as the context and circumstances under which we carried out this project are unique, and our samples are not necessarily representative of the larger population of ASA survivors within the focal state, let alone across state lines. However, there are different types of generalizability, such as when the findings resonate with or ring true to people’s lived experiences (representational or naturalistic generalizability); when the findings can be transferred and easily applied to inform what is happening in other contexts (inferential generalizability, case-to-case generalizability, or transferability); and when the findings highlight specific concepts that are generalizable rather than focusing on the generalizability from a specific sample to a larger population (analytical, vertical generalizability, or idiographic generalizability; Smith, 2018). The survivors in our sample chose carefully to whom they would disclose and overwhelmingly chose not to engage formal systems due in large part to the incompatibility between MR and their needs and desires. This key finding rings true to experiences articulated by taskforce members in early MR discussions; can be transferred and easily applied to inform what is happening in other contexts; and highlights important concepts—specifically, the key concept that MR can and does deter ASA survivors from disclosing or seeking formal help.

Still, additional research on the role and impact of MR in the context of ASA is sorely needed. While the survivors in our sample provided invaluable insight into the role of MR in their post-assault decision making, it is important to diversify who is included in future studies. As Kennedy et al.’s (2012) model makes clear, there is a complex interplay of individual, institutional, community, and societal factors that manifest in unique ways for individuals occupying different sociopolitical intersections. To understand survivors’ experiences more fully, it is important to engage a wider array of survivors across multiple domains.

First, our sample consisted primarily of survivors of adolescent sexual assault who did not receive formal help; future research should strive to learn from survivors who did and did not engage a wider range of providers in the context of MR. This will allow us to understand not only what influences specific decisions, but also how those decisions play out and shape survivors’ post-assault experiences.

Second, while this study focused on adolescent sexual assault, our sample was age-diverse, with participants ranging in age from 15 to 55 years old at the time of their interviews, and anywhere from a few weeks to four decades transpiring between their focal assault and their participation in this study. This may have impacted recall for those who participated many years after their focal assault occurred. On the other hand, it also permitted a different form of reflection by allowing adults to reflect on their choices as adolescents. In this sample, the reflections and sentiments provided by participants were quite consistent. Future research should continue to examine if and how differences in interview timing post-assault influence participants’ beliefs about their help-seeking and its impact on their lives. If such studies were designed longitudinally, they could additionally explore the long-term impacts of MR polices and survivors’ post-assault decisions on their well-being.

Third, while our sample was somewhat racially diverse and gender diverse, it consisted of mostly white cisgender women and entirely of individuals who were harmed in one Northeastern state. It is important to hear from a wider range of survivors from different communities across the United States, and who occupy different locations within intersecting systems of power. This will allow us to more fully understand the role and impact of MR in adolescent survivors’ post-assault decisions and experiences, and how these decisions and experiences intersect with identity, systems of power, and survivorhood. For example, one “Caucasian” survivor in our sample (Participant 123) briefly mentioned how they knew they were “not personally at high risk of police brutality,” yet still did not want to engage them, while another “Black” survivor (Participant 129) made explicit how police “don’t have a great reputation with people of color,” as the police were “here to serve white people and rich people and not” her. Additional intersectional research—with carefully crafted samples that allow for more intentional examinations of intersecting systems of power and how they shape adolescent survivors’ decision-making and experience—is much needed (e.g., see Bailey et al., 2024).

Finally, our sample consists of individual survivors who chose to participate in our study. It is possible that survivors who chose not to reach out to our research team, or otherwise not participate, are systematically different from those who did. This amplifies the need for additional research on this topic that has not yet been adequately explored in prior research.

In addition to centering and hearing directly from a diverse array of survivors, future research could also make use of administrative and other records created and maintained by responding agencies to understand better how these agencies are operating and the extent to which they are centering survivors’ needs in their service provision. Existing records provide an unobtrusive measure of agency and system operations that can provide invaluable insight. As one such example, the broader project in which this study was embedded relied on existing medical and criminal legal records to examine how MR impacted adolescent sexual assault criminal case progression (Shaw et al., 2024). While this single study does not provide all the answers, the key concept that MR can and does deter ASA survivors from disclosing and seeking formal help invites all communities to consider how they can design ASA response policies and practices that truly prioritize the adolescent survivor.

### 4.2. Implications for Policy and Practice

We found that MR deters ASA survivors from disclosing and seeking formal help due in large part to concerns of unwanted involvement of other entities, and more broadly losing control and agency. These findings point to the importance of designing and implementing ASA response policies and practices that protect adolescent survivors’ privacy and allow them to have as much control as possible in deciding what happens next, including who learns about their assault, who is invited to be a part of the response process, and what steps are taken to ensure their safety, meet their needs, and promote their healing and recovery.

Too often, adolescents’ developmental stage is used to justify others having control over them and making decisions on their behalf (e.g., see Bailey et al., 2024; Bell, 1995). Adolescent survivors, like all adolescents, have come to expect this. The survivors we interviewed discussed how they were concerned that if they chose to disclose to certain individuals or engage formal systems, others would take control and the adolescent would not be allowed to decide what would happen next. In all cases of sexual assault, it is critical to support the survivor in exercising agency and reclaiming control to reduce additional harm to the survivor and promote their healing and recovery (e.g., see Campbell et al., 2015; Frazier, 2003; Greeson & Campbell, 2011; Walsh & Bruce, 2011; Zweig & Burt, 2007). This holds for and should be applied to adolescent survivors, too. Their developmental stage and legal status as minors should not be used to undermine their ability to make important decisions and to exercise power.

Instead, individual responders should support adolescents by providing clear information about their options, what is possible, and what they can expect. To the extent possible, responders should allow adolescent survivors to drive all decisions. This requires the responder to be intimately familiar with organizational and legislative policies that require certain responses, including MR laws. The responder must know what is legally required of them and be able to distinguish it from what has simply become a normed practice. If a certain response has become normalized but is not required, it provides an opportunity for the responder to center the adolescent and have them drive what happens next rather than making the decision on their behalf. To maximize adolescents’ decision-making power, it is important to consider decisions, both big and small, and identify any decision points where the adolescent survivor can exercise control. For example, if organizational or legislative policy requires a certain response—what must happen—there might be an opportunity for the adolescent survivor to decide when or how it happens, as wished for by Participant 107 in our study. To do this, responders will have to deliberately unlearn some of their current response patterns (Hislop et al., 2014). They will need to revisit organizational and legislative policies to understand the distinction between required and normed responses, and they will need to slow down to be able to identify hidden decision points that offer the opportunity for survivors to decide what happens next, how, and when.

Beyond the individual, organizations that respond to ASA need to revisit their policies and practices to assess the extent to which they support and allow for adolescent survivors to exercise agency and drive decision-making. Of course, organizations need to ensure that their policies abide by local, state, and federal laws, including MR laws. However, there can be significant variation in how different organizations implement policies to satisfy the same legal requirements. Indeed, there is a high level of variation in how MR laws are implemented at the organizational level (Bailey et al., 2023), suggesting organizational policies are designed to satisfy not only legal requirements but organizational needs, too. This may include the need to provide their employees with additional guidance on precisely how to implement a policy into practice, or the need to protect the organization from being held liable should something go awry. For example, university policies that mandate employees to report student disclosures of sexual assault to university officials have been found to prioritize legal liability and protecting the university over survivor well-being and protecting survivors from additional harm (Holland et al., 2018). The same may be true for organizational policies regarding the implementation of MR laws, meaning that organizations are protecting themselves at the expense of adolescent survivors. ASA survivors want and can make important post-assault decisions. Enabling them to exercise agency in deciding how to proceed following a sexual assault can aid in their recovery and healing, and help ensure they become connected to needed resources. Like the individual responder, organizations should review and assess their policies to identify what responses are required by law, and where there might be opportunities to provide a less prescriptive response in favor of allowing the adolescent survivor to decide what happens next. This, too, will require deliberate organizational unlearning—the discarding of old routines to make way for new ones (Tsang & Zahra, 2008). Organizations should equip their employees with tools, knowledge, and support so they are prepared to work alongside adolescent survivors and support their informed decision-making so that all post-assault decisions are made based on what best serves the survivor, not what best serves the organization.

Beyond the individual and organization, our study also invites communities to reexamine existing legislation on MR, how it applies to ASA, and if revisions or additions are warranted to best meet the unique needs of ASA survivors. Most relevant to the current study, MR laws vary across states regarding the circumstances that require a mandatory report and the specific entities that are included in the MR response (see Bailey et al., 2023). First, in some states, MR legislation is unequivocal in defining and providing an exhaustive list of the specific victim–perpetrator relationships that require a mandatory report. For example, Illinois law requires mandatory reporters to file a report when they have reasonable cause to believe that a child (i.e., under 18 years old) is being abused or neglected. The law further specifies that ‘abuse’ is defined in part by the nature of the relationship between the alleged perpetrator and victim. As codified in the Illinois Abused and Neglected Child Reporting Act, the alleged perpetrator must be a “parent or immediate family member, or any person responsible for the child’s welfare, or any individual residing in the same home as the child, or a paramour of the child’s parents” (325 ILCS 5). If there is any other relationship between the victim and alleged perpetrator, a mandatory report should not be filed. If the case or incident is reported to CPS and the relationship between the victim and alleged perpetrator is not one of those explicitly mentioned, the incident does not qualify as abuse, CPS has no jurisdiction, and the report is not taken (Children’s Justice Task Force, 2020). Other states are less clear in defining the specific victim–perpetrator relationships that meet the definition for abuse. This is critically important in the context of ASA. Unlike younger sexual assault victims, adolescents are most often sexually assaulted by a same-age peer or other non-familial known person (Crawford-Jakubiak et al., 2017; Finkelhor et al., 2014; Giroux et al., 2018; Muram et al., 1995; Peipert & Domagalski, 1994; Stein & Nofziger, 2008). This means that in some states, like Illinois, mandatory reports would not be filed nor accepted by CPS for most ASA cases, allowing adolescents to seek post-assault care and services without fear of others being notified or involved in the response process without their permission. For many of the participants that we interviewed, this may have been enough for them to access needed post-assault care. Of course, this also depends on adolescents being able to consent to receiving care and services independently and without parental consent, as the participants we interviewed were also concerned about their parents being notified. States should examine their MR laws to ensure they provide enough specificity as to what exactly constitutes child abuse so that it aligns with their CPS’s definition of child abuse and what they would ‘screen in’ for a response. There should be a particular focus on defining the nature of the relationship between the victim and perpetrator, as this is often an important criterion that CPS uses to determine if they have jurisdiction and if further investigation or intervention is needed. Informing adolescents about such changes, their rights, and options may result in more adolescent survivors seeking and becoming connected to important post-assault care and services. As states examine their own MR laws and the extent to which adolescents can consent to care on their own, they should look to other states for examples of what might be possible.

Second, states’ MR laws vary in terms of which entities are a part of the response. In this study, the MR response under evaluation involved CPS, parents, police, and prosecution. Some states do not legally require police and prosecution to be notified as part of the MR response (Bailey et al., 2023). In our interviews with survivors of ASA, participants were most concerned with police becoming involved should they choose to disclose or seek formal help. This often resulted in, or contributed to, participants’ decisions not to make certain disclosures. Additionally, we found in the larger mixed-methods project in which this study was embedded that including police and prosecution in the legally required MR response did not result in higher rates of criminal prosecution or convictions (Shaw et al., 2024). This means that such MR responses not only deter survivors from seeking care but do not have their intended impact of increasing prosecution rates. Instead of legally requiring that police and prosecution be notified as part of the MR response, communities should provide criminal legal system engagement as an option to adolescent survivors. This would remove undesired police involvement as a deterrent from seeking post-assault services. If such changes were made, and effectively communicated to adolescents, they may result in more ASA survivors seeking out and receiving the care they need and deserve.

Finally, beyond evaluating specific elements of MR laws related to the circumstances in which they apply and the entities to whom reports must be made, communities should consider the specific purposes of MR policies and examine whether they are realizing their intended impact. While additional research is needed to understand more fully the effects of MR laws, this study, and the broader project in which it was embedded, suggests that MR has the potential to harm those it is intended to help by stripping survivors of their agency and requiring them to walk down paths they did not choose and cannot control. MR policies should not be continued simply because they were designed and implemented with good intent. To best serve survivors, we must revisit how these policies are designed and implemented in the context of ASA, and take seriously the possibility that no matter how well-intentioned, MR policies that dictate a one-size-fits-all response may cause more harm than good. We must be willing to think creatively and collaboratively alongside ASA survivors to ensure they have what they need and deserve to heal and recover after the assault.

## Figures and Tables

**Table 1:** Participant descriptions and mandatory reporting impact category.

ID	Age at Interview	Age at Focal Assault	Race	Gender	MR Impact Category
111	25 years old	17 years old	White	Female	Primary Deterrent
113	22 years old	17 years old	Hispanic or Latino	Non-binary, more female	Primary Deterrent
115	21 years old	13 or 14 years old to 16 or 17 years old	White	Female	Primary Deterrent
126	20 years old	13 or 14 years old	Korean, Asian, Mixed	Transmasculine person	Primary Deterrent
129	35 years old	15 years old	Black	Cisgender female	Primary Deterrent
130	29 years old	13 years old	White	Female	Primary Deterrent
136	37 years old	14 or 15 years old	American descendant of slaves	Woman	Primary Deterrent
139	23 years old	13 and 15 years old	White	Cis-female	Primary Deterrent
141	20 years old	14 years old	White	Female	Primary Deterrent
108	25 years old	16 and 17 years old	White	Cisgender female	Secondary Deterrent
110	55 years old	14 years old	White	Female	Secondary Deterrent
117	20 years old	15 years old	White	Female	Secondary Deterrent
122	26 years old	14 years old	White	Female	Secondary Deterrent
123	19 years old	17 years old	Caucasian	Presenting as a woman but still figuring stuff out	Secondary Deterrent
124	22 years old	15 years old	White Ashkenazi	Gender-fluid, non-binary	Secondary Deterrent
128	32 years old	12 to 14 years old	White	Nonbinary	Secondary Deterrent
107	15 years old	9 to 13 years old	Black	Female	Would-be Deterrent
138	16 years old	16 years old	Black and Puerto Rican	Female	Would-be Deterrent
101	16 years old	15 years old	African American	Female	Nonissue
118	21 years old	17 years old	White	Female	Nonissue
119	35 years old	17 years old	White	Female	Nonissue

## Data Availability

The qualitative interview data collected and reported on in this study are not publicly available as it is impossible to deidentify the data such that it adequately protects the identify and privacy of participants while ensuring it retains its meaning.

## Abbreviations

The following abbreviations are used in this manuscript:

ASA	Adolescent Sexual Assault
CPS	Child Protective Services
MR	Mandatory Reporting
IRB	Institutional Review Board
PI	Principal Investigator
SANE	Sexual Assault Nurse Examiner
